# Tandem keyhole foraminotomy in the treatment of cervical radiculopathy: retrospective review of 35 cases

**DOI:** 10.1186/1749-799X-9-38

**Published:** 2014-05-16

**Authors:** Hidetomi Terai, Akinobu Suzuki, Hiromitsu Toyoda, Hiroyuki Yasuda, Kunikazu Kaneda, Hirofumi Katsutani, Hiroaki Nakamura

**Affiliations:** 1 Department of Orthopaedic Surgery, Osaka City University Graduate School of Medicine, 1-4-3 Asahimachi, Abeno-ku, Osaka 545-8585, Japan; 2 Department of Orthopaedic Surgery, Shimada Hospital, 100-1 Kashiyama, Habikino, Osaka 583-0875, Japan

**Keywords:** Keyhole foraminotomy, Cervical radiculopathy, Microsurgical decompression

## Abstract

**Background:**

There has been no report regarding the results of two-level keyhole foraminotomy. The purpose of this study was to detail clinical outcomes following consecutive two-level cervical foraminotomy (tandem keyhole foraminotomy (TKF)) in patients with radiculopathy.

**Methods:**

The authors conducted a retrospective review of 35 cases involving patients treated by a single surgeon using TKF. Clinical symptoms, data of physical examinations, pathology and clinical outcomes were detailed and discussed about this surgical method.

**Results:**

Patients consisted of cervical disc herniation (CDH) (19/35), cervical spondylotic radiculopathy (CSR) (13/35), and cervical spondylotic amyotrophy (CSA) (3/35). TKF was performed from C3 to C5 in 2 patients (6%), from C4 to C6 in 7 patients (20%), from C5 to C7 in 23 patients (66%), and from C6 to T1 in 3 patients (8%). The mean operative duration was 99.2 min (range, 72 to 168 min). The mean estimated blood loss was 55.8 g (range, 0 to 200 g). Radicular pain was relieved within 3 months in 88% (29/32) and in 97% (31/32) at final follow-up. Resolution of muscle weakness was recognized within 6 months after operation in all CSA cases. Sixty-six percent of patients showed a greater than 20% deficit in grip weakness on the affected side compared with the normal side. After pain was relieved, grip strength improved by more than 15%.

**Conclusions:**

TKF is a safe and highly effective procedure for patients with cervical radiculopathy and does not require invasive preoperative examinations. Further investigation is required to determine the effects of consecutive facetectomy.

## Introduction

Cervical radiculopathy is common among middle-aged to elderly individuals and is usually caused by cervical disc herniation or bone spurring from cervical spondylosis. Symptoms include neck pain, arm pain, motor weakness, and decreased sensory perception, depending on which nerve roots are compressed. Cervical radiculopathy can be treated conservatively or surgically; most cases can be treated with conservative therapy [1], but those that are refractory to conservative therapy are candidates for surgical intervention.

The two main surgical approaches are anterior and posterior. The anterior approach, anterior cervical decompression and fusion (ACDF), is now widely accepted and is performed in many institutions because of ease of exploration and setting; however, there are many disadvantages, including symptomatic adjacent disc disease, postoperative dysphagia [2,3], and the risk of donor-site morbidity. The benefits of posterior decompression for cervical radiculopathy, or laminoforaminotomy, have been demonstrated by several authors [4-6]. One advantage of posterior cervical foraminotomy (PCF) is the avoidance of fusion and preservation of range of motion, which may contribute to lower risk of adjacent disc degeneration. Another advantage is that surgeons can visualize the compressed nerve root directly and confirm the decompression. Because PCF presents limited risks of damage to paravertebral muscles and facet joints [7] and esophagus, we prefer keyhole foraminotomy as PCF for cervical radiculopathy because the procedure is less invasive and because it produces excellent outcomes and lower complication rates [8].

Although there are many reports on PCF, few reports on multilevel keyhole foraminotomy exist. In the present study, we performed two-level keyhole foraminotomy (tandem keyhole foraminotomy (TKF)) in 35 consecutive patients. The purpose of this report is to detail our surgical method and to examine the clinical outcomes following TCF in patients with cervical radiculopathy.

## Methods

### Patient characteristics

Between August 2008 and October 2013, TKF was performed in our department and affiliate hospital on 35 patients with cervical radiculopathy. All patients had received more than 3 months of conservative treatment, including cervical epidural steroid injection. Patients who had myelopathy or past cervical surgery were excluded. Preoperative and perioperative data were obtained from a review of patients' charts and clinical results obtained postoperatively. For each patient, diagnosis (cervical disc herniation (CDH), cervical spondylotic radiculopathy (CSR), cervical spondylotic amyotrophy (CSA)), duration of symptoms, age, sex, neurological findings (muscle weakness, sensory disturbance, deep tendon reflexes, results of Jackson and Spurling tests, and grip strength of both hands) [9], primary cervical level involved, operated levels, operative duration, estimated blood loss, complications, and time to pain relief were obtained from medical records. The patients were followed up until they had been pain-free for more than 1 month or until there was appreciable recovery of muscle strength.

### Selection of surgical levels

Surgical levels were first determined by neurological examinations, then confirmed by magnetic resonance imaging (MRI) and functional X-rays obtained in flexed, neutral, and extended positions of the neck as references. No patient had undergone selective nerve root block or myelography to determine the responsible level. Neurological examinations included manual muscle testing (MMT), sensory loss, and deep tendon reflex. We placed more importance on the neurological examination because MRI alone is not sufficient to determine and confirm the surgical level.

We first determined the primary responsible level. We considered the key muscle of each nerve root to be the following: C5, deltoid; C6, biceps and wrist extensors (WEs); C7, triceps, wrist flexors (WFs), and extensor digitorum communis (EDC); and C8, abductor pollicis brevis (APB) and abductor digiti minimi (ADM). If the patient had weakness in the above-mentioned muscles, we considered the associated spinal level to be responsible. If patient had multilevel muscle weakness, the nerve root associated with the weakest muscle was considered responsible. If two representative muscles showed the same weakness, we considered both to be responsible levels. If there was no muscle weakness, we used area of sensory disturbance as the next most important reference. After the determination of primary responsible level, operative levels were determined. Operative levels were the primary responsible level and its adjacent level, determined by the distribution of sensory loss and the area of radiating pain, as reported by Tanaka et al. [10]. If there was neither muscle weakness nor sensory disturbance, disc degeneration by MRI was used to determine the operative level. The most affected levels were C5-6 and C6-7.

### Surgical technique

**Figure 1:**
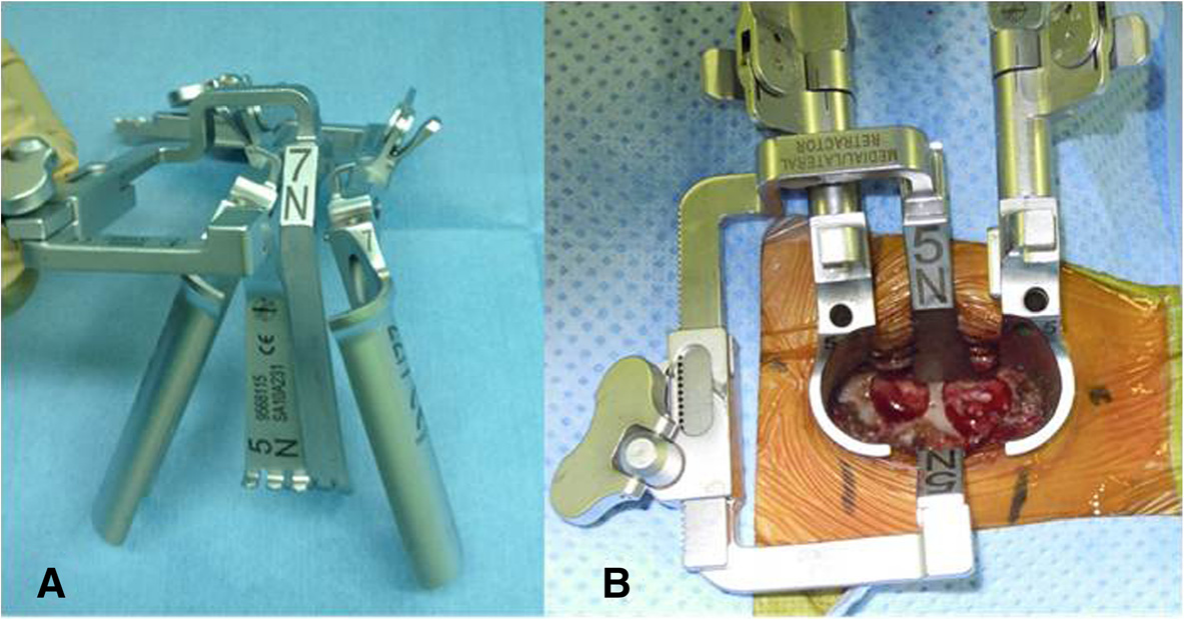
**Retraction system and TKF. (A)** METRx™ *Quadrant.***(B)** Tandem keyhole foraminotomy as seen through the *Quadrant.*

**Figure 2:**
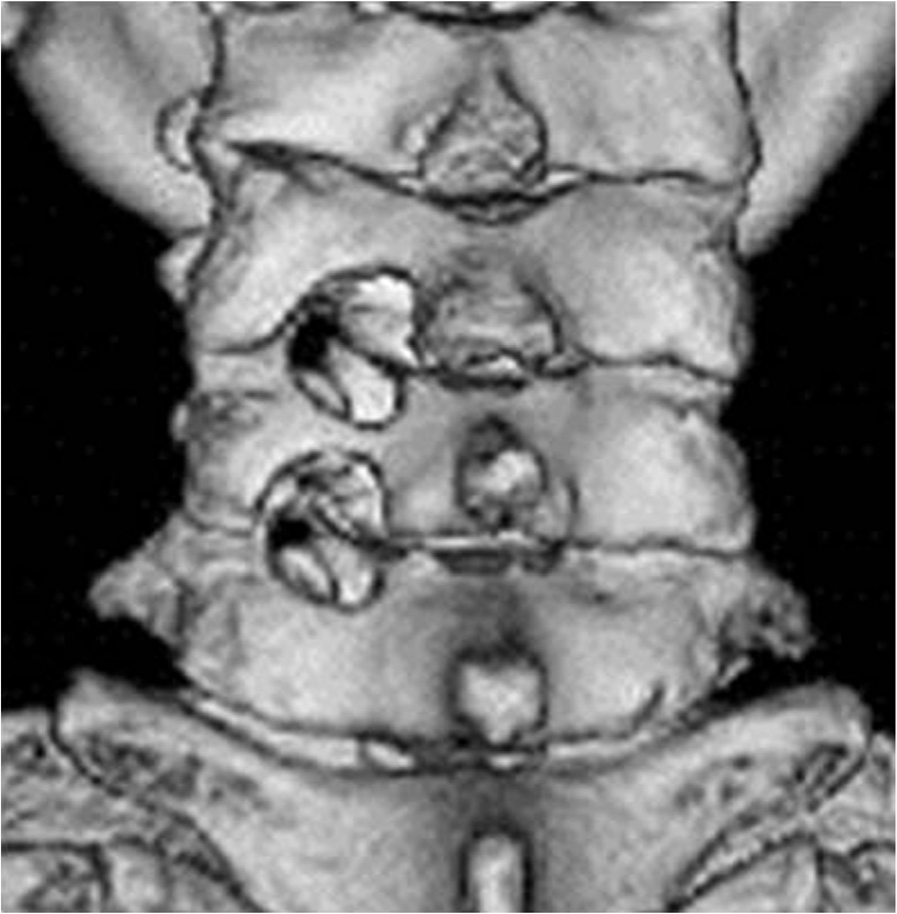
**Postoperative 3D CT view.** Tandem keyhole foraminotomy was performed in C5/6/7. Half of the facet joints were preserved. The lateral border of vertebral body was recognized through the decompressed hole.

**Figure 3:**
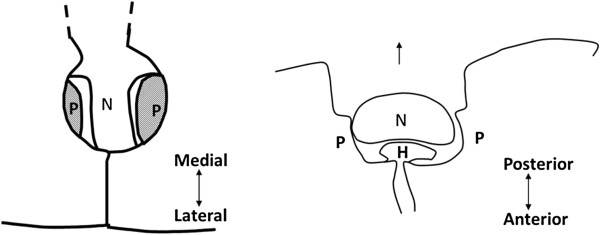
**Scheme of the operative site.** N, nerve root; P, pedicle; H, herniated disc or bony spur. Left: posterior view of the operating field. Decompression must be done until both proximal and distal pedicles were confirmed. Right: nerve root can be shifted posteriorly after appropriate decompression. Herniated discs or bony spur need not to be removed.

All patients were placed prone in reverse Trendelenburg position with the head positioned in a Mayfield fixator. After identifying the operative level using fluoroscopy, a 3- to 3.5-cm incision was made just off the midline at the target level. After subperiosteal dissection of the paravertebral muscle from the lamina, the METRx™ Quadrant system (Medtronic Sofamor Danek, Memphis, TN, USA) was used for retraction (Figure 1). We did not use the intramuscular approach using tubular retractors because of the muscle density in this area, which we feel is too high for a 25-mm-diameter retractor. After re-confirmation of surgical levels with lateral plain X-ray, we performed keyhole foraminotomy at two consecutive levels under the utilization of operative microscope to facilitate illumination and visualization of cervical nerve roots. The keyhole was a circle with a radius half the length of the facet (Figure 2). Decompression was performed until both proximal and distal pedicles were confirmed longitudinally, and a probe was easily inserted into foramen laterally. No soft disc herniation was removed; we believe the pathology of cervical radiculopathy is the impingement, which can be released by removing counterparts of herniation or bony spur, and removal of extruded disc may have a potential risk of excessive retraction causing motor palsy (Figure 3) [11,12].

After the surgical bed was irrigated with normal saline, suction drainage was placed in the area of the resected laminae and kept in place for 24 to 48 h or until drainage was reduced to less than 100 mL/day. A cervical collar was used only a couple of days after surgery.

### Statistical analysis

The Chi square test and Mann-Whitney *U* test were applied for the comparison of pre- and postoperative global results and patient's characteristics. The descriptive assessments and analytical statistics were performed, depending on the group characteristics with the program package SPSS (IBM Co., New York, NY, USA). A positive significance level was assumed at probability of less than 0.05.

## Results

### Patient characteristics and presenting symptoms

Patient characteristics by diagnosis are presented in Table 1. Patient's age of CSA was significantly older than CDH and CSR. CSR was more common in male. Estimated primary responsible levels and operated levels are presented in Table 2. C5/6 (31%), C6/7 (14%), and C5/6/7 (34%) were more common affected levels in this series. Most operated levels were C5/6/7 (66%), followed by C4/5/6 (20%). The mean follow-up period was 6 months. The mean duration of symptom before surgery was 2.9 months (1 to 4 months) in CDH, 3.5 months (1 to 12 months) in CSR, and 7.0 months (1 to 12 months) in CSA.

**Table 1:** Patient's characteristics by diagnosis

	**CDH (*n *= 19)**	**CSR (*n *= 13)**	**CSA (*n *= 3)**
Age (years)	49.4 (29 to 73)	52.8 (33 to 72)	69.3 (64 to 74)
Gender (M:F)*^,^**	12:7	13:0	3:0
Number of patients with muscle weakness	12 (63%)	9 (69%)	3 (100%)
Number of positive Jackson and Spurling tests	19 (100%)	11 (85%)	0 (0%)
Duration of symptoms (months)	2.9 (1 to 4)	3.5 (1 to 12)	7.0 (1 to 12)

*P < 0.05 (significance between CDH vs CSR). **P < 0.05 (significance between CDH/CSR and CSA).

**Table 2:** Estimated primary responsible levels and operated levels (%)

	**Estimated primary responsible level**	**Operated levels**
C3/4	0	
C4/5	4 (11%)	
C5/6	11 (31%)	
C6/7	5 (14%)	
C7/T1	1 (3%)	
C3/4/5	0	2 (6%)
C4/5/6	1 (3%)	7 (20%)
C5/6/7	12 (34%)	23 (66%)
C6/7/T1	1 (3%)	3 (8%)

### Clinical follow-up

One patient required reoperation and debridement because of deep infection due to preexisting severe atopic dermatitis. Another required wound treatment because of incomplete healing of the superficial wound. Radicular pain was completely relieved within 1 month in 11 patients (32%) and within 3 months in 18 patients (51%). One patient (3%) who experienced pain and had negative Jackson and Spurling tests preoperatively did not respond to surgery [13]. One patient who had had negative impingement signs but muscle weakness at preoperative examination recovered muscle strength by 6 months after surgery (Table 3). All patients diagnosed with cervical spondylotic amyotrophy had manual muscle testing level 1 muscle weakness in the deltoid that recovered to level 3 in one patient and level 5 in two patients.

**Table 3:** Time until radicular pain disappeared

	**Number of patients (*n *= 32)**
Less than 1 month	11 (32%)
1 to 3 months	18 (56%)
3 to 6 months	1 (3%)
More than 6 months	1 (3%)
Not responded	1 (3%)

**Figure 4:**
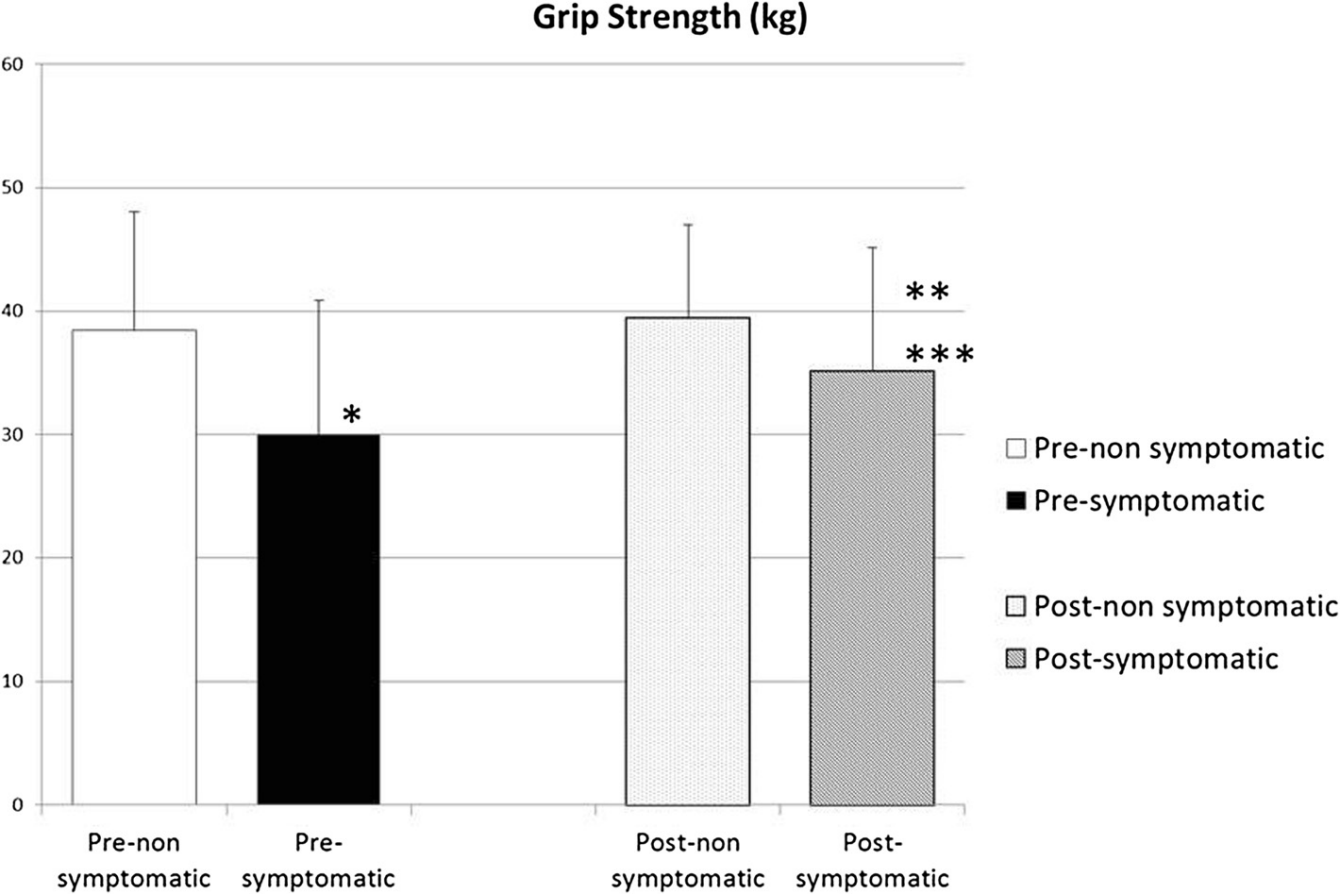
**Grip strength (kg) of pre- and post-operation shown in symptomatic and non-symptomatic side.** *P < 0.01: pre-nonsymptomatic vs pre-symptomatic. **P < 0.01: post-nonsymptomatic vs post-symptomatic. ***P < 0.01: pre-symptomatic and post-symptomatic.

Twenty-three patients (66%) had a greater than 10% deficit in muscle strength compared with the normal side preoperatively (mean, 77.6% of contralateral muscle strength; range, 22.7% to 106.1%). Eighteen patients (51%) still had a more than 10% deficit in grip strength at final follow-up; however, average grip strength improved to 89.3% of the normal side. An average of 15.3% of patients in the improved group had increased grip strength; however, three patients showed a decrease (Figure 4). No patient demonstrated kyphotic or alignment change on cervical plain X-rays at final follow-up.

## Discussion

Cervical radiculopathy is a common condition that produces radiating pain, paresthesias, motor weakness, and/or diminished reflexes associated with impingement of one or more cervical nerve roots. Schoenfeld et al. have reported that the incidence of cervical radiculopathy is 1.79 per 1,000 persons, and individuals older than 40 years, females, and white races in military service are at higher risk [14]. There are many therapeutic options for cervical radiculopathy: surgery, physiotherapy, medication, and a variety of blocks, and treatment vary by doctor. Persson et al. reported that in treating patients with persistent radicular pain, cervical collar, physiotherapy, and surgery are equally effective in the long term [1], and many orthopedists, including spine surgeons, agree with this and never recommend surgery to their patients. However, some patients do require surgery and are satisfied with their results.

Surgical treatment of cervical radiculopathy is divided into two major categories: anterior approach and posterior approach. Anterior approach includes traditional ACDF, artificial disc replacement, and anterior transcorporeal disc resection [15,16]. Postoperative dysphagia and risks of violation of the esophagus or recurrent laryngeal nerve during operation are well-known complications of anterior cervical approaches, although each method can produce good clinical results. ACDF is often performed in young patients; however, ACDF has been shown to accelerate adjacent disc degeneration [17]. In addition, the treatment costs of ACDF and artificial disc replacement are high compared with those of other decompression surgeries [18].

Since the posterior approach was first developed by Northfield in 1955 [19], many surgical modifications have been developed to minimize its surgical invasion and comorbidities such as cervical kyphosis and neck pain [20]. Cervical foraminotomy using a microscope was first reported in 1983 by Williams, who found that 96.5% of patients had resolution of intractable radicular pain within 3 days [5]. In the same year, Henderson et al. also reported clinical details of their 15-year experience of 846 operated radiculopathy cases, in which pain relief was also achieved in 96% of patients [4]. Neither paper reported cervical instability nor major complications such as those seen after ACDF, and their conclusions were that the advantages of posterior foraminotomy were lower complication rates and effectiveness at relieving pain. Our results also showed that 97% of patients could be relieved from pain at final follow-up.

We employed mainly MRI as an imaging modality to determine the responsible level and operative levels. Disc herniation and dural displacement are easily detectable in MRI, but osteophytic nerve root or dural compression is more easily detectable on myelogram and myelo-CT. However, we seldom use myelogram because of toxicity of the contrast agent and patients' potential allergic reactions. It is still difficult to define a lesion using only MRI because MRI cannot provide sequenced slices, although the resolution of MRI is now greatly improved. In cases of multilevel degeneration in particular, it is impossible to decide the responsible level using only MRI and risky to proceed on that basis. For this reason, we employed mainly neurological findings to determine the responsible level. In this series, we obtained functional X-rays at every visit; however, they were used only to check instability pre- and postoperatively. In the reports of both William and Henderson et al., myelogram was performed preoperatively to determine the surgical level, although 76% of patients in series of Williams and 80% of those in the series of Henderson et al. underwent multilevel surgery because of the difficulty of determining the responsible level using only myelogram [4,5].

The primary responsible level in this series was determined by the combination of preoperative neurological examinations such as sensory disturbance, muscle weakness, and tendon reflex [13]. Checking of muscle weakness was more reliable than other tests. Dermatomes of sensory disturbance and tendon reflexes were used as secondary determinants of responsible levels or for reference. Electromyography may be helpful in determining the single responsible level; however, it is not a perfect predictor because each muscle is usually controlled by a number of nerves. Intraoperative use of electromyography and preoperative selective nerve root block are ideal if patients are not averse to the invasiveness of these diagnostic procedures. Henderson et al. reported the accuracy of estimated responsible levels using imaging and preoperative examinations to be 73% for C5-6, 80% for C6-7, and 43% for C7-T1 [4]. Because it can thus be concluded that the responsible level for radiculopathy is not perfectly predicted by neurological examination and image analysis, we employed TKF.

An advantage of TKF is promising clinical results without any invasive preoperative examinations such as myelogram, electromyography, or selective nerve root blocks. Greater than 97% of our patients were relieved of radicular pain or muscle weakness. These results are comparable to those in the reports of Williams and Henderson et al. [4,5]. Another advantage is that we could compare the conditions of two decompressed nerve roots in the same patient. However, further study is required to investigate the accuracy of estimation of the primary responsible level. A disadvantage of TKF may be the additional damage to soft tissues and facet joints at unaffected levels compared with single-level surgery. There was little difference in the size of skin incision and muscle detachment between one or two levels because two adjacent levels are usually in the same operating view when the Quadrant retractor system is used. An attempt should be made to preserve facet joints to prevent cervical deformity, but in this series, only 50% of facet joints were resected and no deformities were observed after operation. However, until longer follow-up study is accomplished, this result is not conclusive.

It is noteworthy that 66% of patients showed a greater than 20% deficit in grip strength on the affected side compared with the normal side. Grip strength weakness cannot be explained by nerve root irritation due to single-level radiculopathy; however, it is well recognized clinically [21,22]. We showed that grip strength improved by more than 15% when pain was relieved. In our analysis in the present study, we did not consider hand dominance.

The limitation of this study was the short-term follow-up period. Degeneration of operated and adjacent levels, recurrence rates, and changes of cervical alignments should be evaluated and discussed in further studies.

## Conclusions

TKF is a safe and highly effective procedure for patients with cervical radiculopathy and requires no invasive preoperative examination. Further investigation into the effects of consecutive facetectomy is required.

## Competing interests

The authors declare that they have no competing interests.

## Authors’ contributions

HTe made substantial contributions to the conception, design, and acquisition of data. AS and HTo assisted in the surgery and in analyzing the statistical data. KK and HK participated in the surgery and in collecting the data at the outpatient clinic. HY and HN checked the manuscript and advised to the revise its content. HTe conceived the study and participated in its design and coordination. All authors read and approved the final manuscript.

## References

[B1] Persson LC Carlsson CA Carlsson JY Long-lasting cervical radicular pain managed with surgery, physiotherapy, or a cervical collar. A prospective, randomized study Spine (Phila Pa 1976) 1997 22 7 751 758 10.1097/00007632-199704010-00007 9106315

[B2] Lundine KM Davis G Rogers M Staples M Quan G Prevalence of adjacent segment disc degeneration in patients undergoing anterior cervical discectomy and fusion based on pre-operative MRI findings J Clin Neurosci 2014 21 1 82 85 10.1016/j.jocn.2013.02.039 24035205

[B3] Fengbin Y Xinwei W Haisong Y Yu C Xiaowei L Deyu C Dysphagia after anterior cervical discectomy and fusion: a prospective study comparing two anterior surgical approaches Eur Spine J 2013 22 5 1147 1151 10.1007/s00586-012-2620-5 23277296 PMC3657041

[B4] Henderson CM Hennessy RG Shuey HM Jr Shackelford EG Posterior-lateral foraminotomy as an exclusive operative technique for cervical radiculopathy: a review of 846 consecutively operated cases Neurosurgery 1983 13 5 504 512 10.1227/00006123-198311000-00004 6316196

[B5] Williams RW Microcervical foraminotomy. A surgical alternative for intractable radicular pain Spine (Phila Pa 1976) 1983 8 7 708 716 10.1097/00007632-198310000-00005 6320471

[B6] Zeidman SM Ducker TB Posterior cervical laminoforaminotomy for radiculopathy: review of 172 cases Neurosurgery 1993 33 3 356 362 10.1227/00006123-199309000-00002 8413864

[B7] Jagannathan J Sherman JH Szabo T Shaffrey CI Jane JA The posterior cervical foraminotomy in the treatment of cervical disc/osteophyte disease: a single-surgeon experience with a minimum of 5 years' clinical and radiographic follow-up J Neurosurg Spine 2009 10 4 347 356 10.3171/2008.12.SPINE08576 19441994

[B8] Riew KD Cheng I Pimenta L Taylor B Posterior cervical spine surgery for radiculopathy Neurosurgery 2007 60 1 Supp1 1 S57 S63 17204887 10.1227/01.NEU.0000215409.64026.E2

[B9] De Luigi AJ Fitzpatrick KF Physical examination in radiculopathy Phys Med Rehabil Clin N Am 2011 22 1 7 40 10.1016/j.pmr.2010.10.003 21292143

[B10] Tanaka Y Kokubun S Sato T Cervical radiculopathy and its unsolved problems Current Orthopedics 1998 12 1 1 6 10.1016/S0268-0890(98)90001-9

[B11] Kang MS Choi KC Lee CD Shin YH Hur SM Lee SH Effective cervical decompression by posterior cervical foraminotomy without discectomy J Spinal Disord Tech 2013 doi:10.1097/BSD.0b013e3182a35707 10.1097/BSD.0b013e3182a35707 23897055

[B12] Choi KC Ahn Y Kang BU Ahn ST Lee SH Motor palsy after posterior cervical foraminotomy: anatomical consideration World Neurosurg 2013 79 2 405 e1-4 22079277 10.1016/j.wneu.2011.03.043

[B13] Rubinstein SM Pool JJ van Tulder MW Riphagen II de Vet HC A systematic review of the diagnostic accuracy of provocative tests of the neck for diagnosing cervical radiculopathy Eur Spine J 2007 16 3 307 319 10.1007/s00586-006-0225-6 17013656 PMC2200707

[B14] Schoenfeld AJ George AA Bader JO Caram PM Jr Incidence and epidemiology of cervical radiculopathy in the United States military: 2000 to 2009 J Spinal Disord Tech 2012 25 1 17 22 10.1097/BSD.0b013e31820d77ea 21430568

[B15] Choi G Arbatti NJ Modi HN Prada N Kim JS Kim HJ Myung SH Lee SH Transcorporeal tunnel approach for unilateral cervical radiculopathy: a 2-year follow-up review and results Minim Invasive Neurosurg 2010 53 3 127 131 10.1055/s-0030-1249681 20809454

[B16] Sekhon LH Ball JR Artificial cervical disc replacement: principles, types and techniques Neurology India 2005 53 4 445 450 10.4103/0028-3886.22611 16565536

[B17] Wigfield C Gill S Nelson R Langdon I Metcalf N Robertson J Influence of an artificial cervical joint compared with fusion on adjacent-level motion in the treatment of degenerative cervical disc disease J Neurosurg 2002 96 1 Suppl 17 21 11795709 10.3171/spi.2002.96.1.0017

[B18] Bhadra AK Raman AS Casey AT Crawford RJ Single-level cervical radiculopathy: clinical outcome and cost-effectiveness of four techniques of anterior cervical discectomy and fusion and disc arthroplasty Eur Spine J 2009 18 2 232 237 10.1007/s00586-008-0866-8 19132413 PMC2899334

[B19] Northfield DW Diagnosis and treatment of myelopathy due to cervical spondylosis Br Med J 1955 2 4954 1474 1477 10.1136/bmj.2.4954.1474 13269895 PMC1981414

[B20] Scoville WB Whitcomb BB Lateral rupture of cervical intervertebral disks Postgrad Med 1966 39 2 174 180 5903648 10.1080/00325481.1966.11696921

[B21] Suzuki A Misawa H Simogata M Tsutsumimoto T Takaoka K Nakamura H Recovery process following cervical laminoplasty in patients with cervical compression myelopathy: prospective cohort study Spine (Phila Pa 1976) 2009 34 26 2874 2879 10.1097/BRS.0b013e3181bb0e33 19949366

[B22] Joghataei MT Arab AM Khaksar H The effect of cervical traction combined with conventional therapy on grip strength on patients with cervical radiculopathy Clin Rehabil 2004 18 8 879 887 10.1191/0269215504cr828oa 15609843

